# The effectiveness of a sustained nurse home visiting intervention for Aboriginal infants compared with non-Aboriginal infants and with Aboriginal infants receiving usual child health care: a quasi-experimental trial - the Bulundidi Gudaga study

**DOI:** 10.1186/s12913-018-3394-1

**Published:** 2018-08-03

**Authors:** Lynn Kemp, Rebekah Grace, Elizabeth Comino, Lisa Jackson Pulver, Catherine McMahon, Elizabeth Harris, Mark Harris, Ajesh George, Holly A. Mack

**Affiliations:** 1 Translational Research and Social Innovation (TReSI), School of Nursing and Midwifery, Western Sydney University, Locked Bag 1797, Penrith, NSW 2751 Australia; 2 Centre for Health Equity Training Research and Evaluation, part of the Centre for Primary Health Care and Equity, UNSW Australia, Sydney, NSW 2052 Australia; 3 Ingham Institute for Applied Medical Research , Liverpool Hospital Locked Bag 7103, Liverpool BC, NSW 1871 Australia; 4 Department of Educational Studies, Macquarie University, Room 234, X5B, Sydney, NSW 2109 Australia; 5 Centre for Primary Health Care and Equity, UNSW Australia, Sydney, NSW 2052 Australia; 6 Office of the Pro Vice Chancellor, Engagement & Aboriginal & Torres Strait Islander Leadership, Western Sydney University, Locked Bag 1797, Penrith, NSW 2751 Australia; 7 School of Medicine, UNSW Australia, Sydney, NSW 2052 Australia; 8 Psychology Department, Centre for Emotional Health, Macquarie University, Level 7, Room 715, C3A Building, North Ryde, NSW 2109 Australia; 9 Centre for Oral Health Outcomes & Research Translation (COHORT), Western Sydney University, South Western Sydney Local Health District, Locked Bag 7103, Liverpool BC, NSW 1871 Australia; 10 Faculty of Dentistry, University of Sydney, Sydney, NSW 2006 Australia; 11 Faculty of Health, University of Technology Sydney, Ultimo, NSW 2007 Australia

**Keywords:** Australian Aboriginal families, Home visiting, Early intervention, Child development, Community health services, Perinatal care, Postnatal care, Primary prevention, Parenting education, MECSH program

## Abstract

**Background:**

In Australia there is commitment to developing interventions that will ‘Close the Gap’ between the health and welfare of Indigenous and non-Indigenous Australians and recognition that early childhood interventions offer the greatest potential for long term change. Nurse led sustained home visiting programs are considered an effective way to deliver a health and parenting service, however there is little international or Australian evidence that demonstrates the effectiveness of these programs for Aboriginal infants. This protocol describes the Bulundidi Gudaga Study, a quasi-experimental design, comparing three cohorts of families from the Macarthur region in south western Sydney to explore the effectiveness of the Maternal Early Childhood Sustained Home-visiting (MECSH) program for Aboriginal families.

**Methods:**

Mothers were recruited when booking into the local hospital for perinatal care and families are followed up until child is age 4 years. Participants are from three distinct cohorts: Aboriginal MECSH intervention cohort (Group A), Non-Aboriginal MECSH intervention cohort (Group B) and Aboriginal non-intervention cohort (Group C). Eligible mothers were those identified as at risk during the Safe Start assessment conducted by antenatal clinic midwives. Mothers in Group A were eligible if they were pregnant with an Aboriginal infant. Mothers in Group B were eligible if they were pregnant with a non-Aboriginal infant. Mothers in Group C are part of the Gudaga descriptive cohort study and were recruited between October 2005 and May 2007. The difference in duration of breastfeeding, child body mass index, and child development outcomes at 18 months and 4 years of age will be measured as primary outcomes. We will also evaluate the intervention effect on secondary measures including: child dental health; the way the program is received; patterns of child health and illness; patterns of maternal health, health knowledge and behaviours; family and environmental conditions; and service usage for mothers and families.

**Discussion:**

Involving local Aboriginal research and intervention staff and investing in established relationships between the research team and the local Aboriginal community is enabling this study to generate evidence regarding the effectiveness of interventions that are feasible to implement and sustainable in the context of Aboriginal communities and local service systems.

**Trial registration:**

Australian New Zealand Clinical Trials Registry ACTRN12616001721493 Registered 14 Dec 2016. Retrospectively registered.

## Background

Indigenous children in Australia have some of the poorest health and welfare outcomes in the OECD [1]. In Australia there is a commitment to developing interventions that will ‘Close the Gap’ between the health and welfare of Indigenous and non-Indigenous Australians [2] and recognition that early childhood interventions offer the greatest potential for long term change [3]. Early childhood home visiting has been adopted as a key strategy. However the applicability and effectiveness of home visiting interventions has not been demonstrated with Indigenous families, particularly in urban environments.

Over the past decades, a number of interventions have been developed to improve birth outcomes for Indigenous infants. For example, in the Strong Women Strong Babies Strong Culture program in the Northern Territory senior community women visited pregnant Indigenous women to improve attendance at antenatal services and maternal health knowledge and behaviour, with mixed results in four communities [4, 5]. The Ngua Gundi program in Rockhampton, Queensland, provided antenatal home visiting, some postnatal home visiting and support for postnatal groups [6], and whilst there is evaluation evidence that the women liked the program, outcomes have not been published. Also in Queensland the evaluation of the Townsville Mums and Bubs program [7] provides an integrated model of antenatal shared care and has demonstrated improved early presentation for antenatal care, increased number of antenatal visits, and reduced rate of pre-term births, however, birth weight and rates of perinatal mortality were unchanged. The NSW Aboriginal Maternal Infant Health Strategy [8] employs a midwife and Aboriginal Health Worker to provide community-based antenatal services for Aboriginal women, and has demonstrated results similar to the Townsville program. In contrast to the antenatal focus of these programs, the South Australian Family Home Visiting program includes targeting of inter alia families of Indigenous infants and commences postnatally, continuing to child-age 2 years. This service, delivered by mainstream (non-Indigenous) child health nurses has been shown to be acceptable to families and achieved some positive outcomes in parent feelings of attachment and parental role satisfaction [9]. None of these programs, however, provides the continuity of care through pregnancy, infancy and early childhood that has been demonstrated to be effective in trials of sustained nurse home visiting (SNHV) in non-Indigenous populations [10].

Home visiting programs comprising intensive, structured and sustained visits by professionals (usually nurses) commencing antenatally and continuing over the first two years of life show promise in promoting child health and family functioning, and ameliorating disadvantage. When supported by SNHV, trials (predominantly overseas with non-Indigenous communities) have shown a positive effect on parenting attitudes and behaviours and on child cognitive and socioemotional outcomes [11]. This international evidence, however, comes mainly from tightly controlled efficacy trials; much less has been documented about the effectiveness of such programs in practice in the Australian context.

SNHV is now a key government strategy to ‘Close the Gap’ in life expectancy between Indigenous and non-Indigenous Australians with both State and Federal governments investing in improved maternal and child health services for Indigenous families. For example, the Federal Government has invested over $37 million in implementing the Nurse Family Partnership model [12] for Aboriginal families, which has been subject to a formative evaluation only. There is little international or Australian evidence that demonstrates the effectiveness of SNHV for Indigenous infants.

The Maternal (formerly Miller) Early Childhood Sustained Home-visiting (MECSH) program is an Australian-developed structured program of sustained nurse home visiting commencing antenatally and continuing through to child-age 2 years. MECSH is focused on: children’s health and development; parental aspirations for themselves and their child/ren, and a structured child development parent education program. A key strategy is continuity of the nurse home visitor. A randomised trial of the MECSH program, delivered by child health nurses within the context of existing community health service structures, has demonstrated effectiveness in improving the health, health behaviours and the quality of the home environment for children’s development of mothers assessed antenatally as having risk of poorer maternal and/or child outcomes living in a disadvantaged multicultural community in south western Sydney, New South Wales (NSW), Australia. Seventeen (8.5%) of the children in the MECSH trial were from Aboriginal families (note: ‘Aboriginal’ is the preferred term for description of Indigenous persons in NSW). The mothers of Aboriginal children who received the MECSH intervention were highly satisfied with the program, had a rate of retention in the study equivalent to mothers of non-Aboriginal children, and, although not statistically demonstrable, reported positive outcomes in duration of breastfeeding and child development at 18 months compared with Aboriginal mothers in the comparison, non-intervention group. These data suggest that the MECSH program may be an appropriate strategy to improve the health and development of Aboriginal children. The current study is applying this experience gained from the MECSH study, together with the knowledge of the issues for families of Aboriginal infants in an urban community gained from the Gudaga cohort study [13], to determine whether SNHV is effective in “Closing the Gap” for urban Aboriginal families, measuring both proximal outcomes during and at the conclusion of the intervention at child age 2 years, and also longer-term post-intervention outcomes during the pre-school years.

This study is thus generating Australian evidence regarding the effectiveness of interventions that are feasible to implement and sustainable in the context of the local service systems. The interventions aim to reduce the impact of social and environmental factors predisposing urban Aboriginal infants and children to ill health and reducing their life potential. This study is the first study internationally to examine the immediate and longer-term effectiveness of a comprehensive SNHV program commencing antenatally and continuing to child-age 2 years for families of Indigenous infants, with follow-up to child age 4 years.

### Research question and hypotheses

#### Primary research question

What are the differences in length of time breastfeeding, child development at 18 months, and child body mass index (BMI) and developmental outcomes at 4 years of age between Aboriginal children of vulnerable mothers (at risk of poorer maternal and child health and development outcomes) receiving SNHV (Group A) and

- non-Aboriginal children of a matched contemporary cohort of vulnerable mothers receiving SNHV (Group B)?
- an historical cohort of Aboriginal children of vulnerable mothers who did not receive SNHV intervention (Group C)?

#### Hypotheses

1. There will be no significant differences in the primary outcomes between Aboriginal children of vulnerable mothers receiving SNHV (Group A) and non-Aboriginal children in a matched contemporary cohort receiving SNHV (Group B).
2. There will be a difference between Aboriginal children of vulnerable mothers receiving SNHV (Group A) and Aboriginal children in the historical cohort who did not receive SNHV (Group C) of more than:
  (i) 5 weeks duration of breastfeeding, and
  (ii) 5 points in the age standardised Griffiths [14] Quotient (GQ) measure of child development at 18 months compared with Group C GQ measured at 12 months.
  (iii) 15% difference in proportion of children overweight/obese at 4 years.
  (iv) 5 points difference in the Griffiths Quotient (GQ) at 4 years; and 0.5SD difference in child vocabulary development at 4 years.

#### Secondary research questions

1. What are the differences in program implementation for, and the way the program is received by mothers and families of Aboriginal and non-Aboriginal children receiving SNHV?
2. What are the patterns of health and illness for Aboriginal compared with non-Aboriginal children of mothers receiving SNHV, and compared with Aboriginal children who do not receive intervention?
3. What are the patterns of maternal health and health knowledge and behaviours, family and environmental conditions and service usage for mothers and families of Aboriginal compared with non-Aboriginal children receiving SNHV, and compared with Aboriginal children who do not receive intervention?

## Methods/Design

### Setting

The study is being conducted in the Macarthur region of south-western Sydney, Australia. The Macarthur region (Campbelltown, Camden and Wollondilly local government areas) has one of the largest Aboriginal populations in New South Wales (NSW) comprising 8337 people: 3.3% of the regional population of 254,219 and 4.0% of the state Aboriginal population [15].

### Conceptual framework

The study applies an ecological framework, recognising that the health, development and wellbeing of children is the product of complex interacting factors at the individual, family and community level [16]. Interventions that seek to achieve the outcome of healthier children need to also address the health of parents (particularly mothers), family and social functioning, and the environment. The intervention and research also recognises Aboriginal frameworks for reciprocity, respect, equality and responsibility; recognising that the researchers, SNHV program providers and the families all contribute; and that contribution is respected, recognised and valued. The study is conducted in a culturally safe environment through the involvement of Aboriginal research and intervention staff and behaviour that maintains coherence of Aboriginal values and cultures [17, 18], and investment in established relationships between the research team and the local Aboriginal community [19].

### Trial design

The study uses a quasi-experimental design comparing three cohorts of families to explore the effectiveness of SNHV for Aboriginal families. The study design recognises that the use of a randomised control group design is inappropriate in the context of this research. There was apprehension within the Aboriginal community about randomisation associated with equity and the investigators wished to demonstrate a respect for the views of the local Aboriginal community [18], based on discussions with local Aboriginal representatives.

### Participants, eligibility criteria and recruitment

#### Eligibility and recruitment of intervention groups (Groups A and B)

In this study, eligible mothers were those identified as at risk using criteria identified through the responses given by the expectant mother at the routine Safe Start [20] assessment conducted by antenatal clinic midwives for all mothers booking into the local hospital for perinatal care. Mothers in Group A were eligible if they were pregnant with an Aboriginal infant, that is, they or the baby’s father identified as an Aboriginal person, and had one or more of the following vulnerability factors identified from the routine assessment:

- maternal age under 20 years;
- unsupported parent determined as those mothers who were not married or living with a partner;
- late antenatal care (after 20 weeks);
- major stressors in the past 12 months determined by a positive response to the question “Have you had any major stressors, changes or losses recently?”;
- current or history of mental health problem or disorder determined by the mother reporting current or past treatment for emotional problems;
- current probable psychosocial distress determined by an Edinburgh Depression Scale [21] score of 10 or more;
- relationship issues with the mother’s parents if they report that they were hurt or abused as a child in any way;
- current substance misuse determined by a positive response to questions about the use of prohibited substances and/or alcohol;
- history of domestic violence based on reports that they get so angry that they hit or hurt their partner, that their partner or anyone else hits them, hurts them or makes them afraid.

Mothers in Group B were eligible if they were pregnant with a non-Aboriginal infant, that is, neither they nor the baby’s father identified as an Aboriginal person, and had one or more of the vulnerability factors described above. In order to match the demographic profile of Groups A and B, the recruitment of Group B commenced after Group A, with the intent of, as much as possible, matching participants on age and suburb of residence: Group A was recruited between October 2011 and March 2013, and Group B between January 2013 and December 2013.

Mothers who had insufficient English-language proficiency to undertake the antenatal risk assessment in English (that is, those who required the use of a translated assessment instrument or an interpreter) were ineligible to participate. All mothers in the area not eligible to be recruited to participate in the study or eligible mothers who declined to participate received usual care.

Contact details of all eligible mothers were collected by the Senior Research Officer on a weekly basis and entered onto a database. The project officer then telephoned each eligible mother to ask for verbal consent to visit them at home to explain the study in detail and written consent to participate in the study was obtained. The demographic and risk profile of the three study groups is presented in Table 1.

**Table 1:** Demographic and vulnerability profile

	Group A	Group B	Group C
Demographic (*n*)	149	80	132
Mean age (SD) at parturition	25.97 (6.17)	27.96 (7.10)	25.39 (6.27)
Median age (years)	25.5	27.5	24.3
Age range (years)	15–44	16–41	16–42
First time mother *n* (%)	51 (34.2)	38 (47.5)	43 (32.6)
Living in lowest Socio-Economic Index for Areas (SEIFA) *n* (%)	77 (51.7)	28 (35.0)	79 (59.8)
Vulnerabilities at maternity booking (Safe Start assessment)			
Age < 20 years *n* (%)	29 (19.5)	11 (13.8)	35 (26.7)
Not married or living with partner *n* (%^a^) (Data missing: A = 5%, B = 6%, C = 10%)	34 (22.8)	17 (21.3)	72 (54.5)
Late antenatal care *n* (%^a^) (Data missing: A = 3% B = 15% C = 20%)	41 (27.5)	26 (32.5)	31 (23.5)
Major stressor *n* (%^a^) (Data missing: A = 4% B = 6% C = 7%)	77 (51.7)	71 (88.8)	52 (39.4)
Mental health issue requiring treatment *n* (%^a^) (Data missing: A = 3% B = 20% C = 10%)	71 (47.7)	49 (61.3)	31 (23.5)
EDS ≥10 at booking *n* (%^a^) (Data missing: A = 4% B = 2.5% C = 14%)	34 (22.8)	43 (53.8)	26 (19.7)
Abused as child *n* (%^a^) (Data missing: A = 7% B = 48% C = 8%)	45 (30.2)	15 (18.8)	32 (24.2)
Substance misuse *n* (%^a^) (Data missing: A = 79% B = 9% C = 11%)	17 (11.5)	8 (10.3)	17 (12.9)
Family violence *n* (%^a^) (Data missing: A = 34% B = 41% C = 8%)	6 (4.0)	10 (12.5)	26 (19.7)
Number of risks, mean (SD)	2.49 (1.36)	3.29 (1.37)	2.57 (1.61)
Risk range	1–6	1–7	1–7
Median number of risks	2	3	2

^a^Presence of vulnerability recorded as percentage of total group N

#### Eligibility and recruitment of Aboriginal non-intervention group (Group C)

The Gudaga cohort (*n* = 149) was recruited between October 2005 and May 2007. Recruitment was conducted in the postnatal ward of Campbelltown Hospital [13]. All infants whose mothers identified them as having an Aboriginal mother or father were eligible to participate. The subset of families in the Gudaga cohort who were assessed at the routine assessment by midwives antenatally as having one or more of the above listed vulnerability factors are included in the historical non-intervention group (*n* = 132).

### Sample size in relation to study effect sizes

Recruitment of 149 participants (Group A) and 80 (Group B) was achieved. A sample size of 75 participants per intervention Group A, Group B and 132 participants in the non-intervention Group C has power of 0.80 at the 95% level to detect a 5 point difference in the Griffiths Quotient (GQ), and a 5 week longer duration in breastfeeding.

- The effect size of 5 weeks duration in breastfeeding is based on the Group C data that showed at 2–3 weeks postnatally (*n* = 122) only 38.6% of mothers were still breastfeeding. The MECSH study demonstrated that mothers of Aboriginal children who received the SNHV intervention breastfed for an average of 14 weeks compared with 8 weeks for mothers of Aboriginal children in the non-intervention control group.
- Results for the broader Gudaga cohort show that 36.9% of children were overweight/obese at 24 months [22]. Evidence suggests that the proportion of overweight/obese children in the cohort will rise in the preschool years [23]. It is expected that the intervention will reduce the proportion of overweight/obesity by at least 17% in the immediate prior-to-school period.
- The effect size of 5 points in the GQ is also based on data from the Group C cohort that showed a mean GQ of 95.35 (SD 10.4) compared with the population norm mean of 100.5 (SD 11.8) and also unpublished data from the MECSH study that demonstrated that Aboriginal children who received the SNHV intervention (*n* = 7) reported a mean mental development score of 101.0 compared with 96.3 for Aboriginal children in the non-intervention control group.

### Retention and attrition

**Fig. 1:**
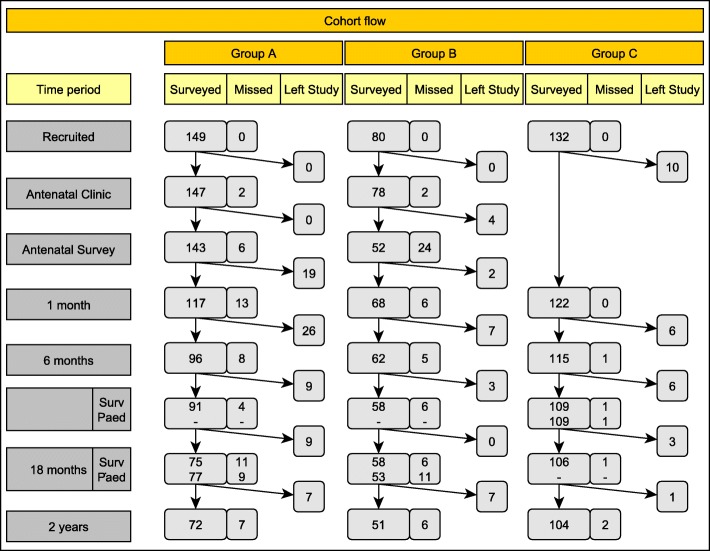
Recruitment and retention flowchart

The retention of participants in the trial to child-age 2 years is presented in Fig. 1. Retention of Group C to child age 4 years was 75% (*n* = 99). Active retention strategies have been and continue to be in place including: asking mothers to provide the name and phone number of two relatives or friends who could be contacted by the researchers to reach them; providing mothers with details of the research team to advise of changes in contact details; sending thank you notes and relevant holiday greeting cards to all participants [24]; using project officers experienced in interviewing families in their own home and trained in the sensitive administration of the data collection tools. Importantly, the project officers employed to work with the Aboriginal families (Group A) are local Aboriginal women and all project officers wear a uniform clearly identifying them with the program. Each time a data collection visit is made a health pack including a small gift for the mother and baby is provided as a thank you for her time. The small gifts included in the health packs are specifically designed to engender a sense of involvement in the project. Further, the research team work closely with the local Aboriginal community through the Aboriginal community controlled medical service, the local community chose an Aboriginal name for the program (Bulundidi Gudaga, meaning happy baby from healthy pregnancy in the local language), and information about the program was and continues to be extensively disseminated within the community.

### Intervention

#### Intervention groups (Groups A and B)

The intervention groups received the MECSH program [25] provided by the local public community health service. The program consisted of at least 25 home visits (actual number of visits determined by need) primarily by the same program nurse, commencing at (on average) 26 weeks gestation and continuing through the first 2 years post birth. The nurse was supported by a social worker, and all nurses working with Aboriginal families were also supported by Aboriginal Health Workers. All intervention staff (nurses, Aboriginal health workers and social worker) received additional training in the program model and cultural competency. The MECSH program home visits were standardised as follows:

- A minimum of three antenatal home visits and postnatal visits within one week of birth, and then at least weekly until 6 weeks; second weekly until 12 weeks; monthly to 6 months; bi-monthly until 2 years. The content of each home visit was individually tailored to the mother’s needs, skills, strengths, capacity, and cultural needs.
- Structured child development parent education program: Parents as Teachers [26].
- Explicit strategies to facilitate access to Aboriginal and non-Aboriginal early childhood health services, volunteer home visiting services and family support services within the Macarthur area.
- Encouragement for families to attend group activities and link into community activities in the local area.

#### Historical Aboriginal non-intervention group (Group C)

The non-intervention group received usual care for families in the Macarthur area, that is:

- Antenatal care according to NSW Health maternity care and Safe Start [20] guidelines and protocols;
- One postnatal home visit by a nurse from the regular child and family nursing service (within 2 weeks of baby’s birth);
- Additional postnatal home or clinic visits with the regular child and family nursing service as indicated by protocols in usual care;
- Volunteer home visiting services and family support services within the local area, as available.

Hence the key differences in the intervention were: home visiting commencing antenatally; continuity of care by the same nurse throughout the 2½ year program; care provided by nurses with additional individualised training in the program model; standardised structured antenatal and postnatal home visiting program to the child’s second birthday; dedicated social worker; dedicated Aboriginal Health Workers (Group A only); structured child development parent education program; group activities and proactive strategies to establish links to community activities.

### Program adaptation, implementation and monitoring

The MECSH program was adapted to provide a culturally appropriate intervention for Aboriginal families. In 2008, a Health Impact Assessment (HIA) [27] was undertaken in consultation with the local Aboriginal community, the Aboriginal medical service and the community health service, to identify and mitigate any aspects of the MECSH intervention that may impact negatively on Aboriginal families. For example, the HIA recommended that an Aboriginal Health Worker be present at the initial contact between the nurse and each Aboriginal family, and continue to visit and support the families and nurses throughout the intervention.

Program documentation (minutes of staff meetings, procedures and protocols, training) were continually reviewed to identify: barriers and facilitators to the uptake and retention in the intervention program, cultural adaptation, workforce skills training, and monitoring of supervision needs and workload; actively promoting links between health home visiting, child protection services, and non-government agencies. Nurses completed checklists on completion of each home visit, detailing the interventions and tasks undertaken. The collated data were used to identify the content of interventions related to the age of the child for Aboriginal and non-Aboriginal families, and to provide ongoing quality feedback to the intervention team.

### Data collection, management and analysis

Data collection by trained project officers is undertaken at recruitment antenatally (Groups A and B only), and then at 1, 6, 12, 18, 24, 36 and 48 months postnatally. On each occasion, a 30 min face-to-face questionnaire is completed by the child’s primary carer (usually mother). At 18 and 48 months the children attend a health and development assessment conducted by a paediatrician at the local hospital. Where needed, transport to the hospital or a home-based assessment is provided. At 48 months the children’s dental health will be assessed by a trained dental therapist at the local community health centre or the child’s home.

### Data coding, security and monitoring

The participants’ identity is protected by assigning a unique ID number to each primary carer/child dyad. Questionnaire data were recorded on paper forms for surveys up to 18 months with all paper based information filed under the participants ID number in locked filing cabinets in a secure building. Questionnaire data for 18 months onwards are recorded electronically using a secure online data entry platform and database with the data entered via tablets on site by the project officers in collaboration with the parents. The online platform provides data collection, backup and security services with the data collected and recorded using study ID numbers only. Health, development and dental assessment data are scored by the assessor on paper forms and stored in a secure building. All study data are input into a SPSS database and stored on a secure, password protected network accessible only by research staff. The study did not employ a data monitoring committee as the intervention has minimal risks. Data monitoring was conducted with the project officers reporting weekly to the project manager with any issues for discussion and documentation.

### Critical events

At each contact the occurrence of any of the following events is noted: maternal death, child death, or child placement in out-of-home care. Descriptive analyses will be undertaken as the incidence of these events is low in the Australian population.

### Outcomes

Primary and secondary outcomes are detailed in Table 2. Where appropriate, measures have been validated and/or benchmarked against the routine data collections of NSW Health (Child Health Survey) and national data collections (such as National Perinatal Statistics).

**Table 2:** Study measures

		Measures	Rationale	Collection Schedule								Instrument
					Postnatal Month							
				A	1	6	12	18	24	36	48	
Primary Outcomes		Breastfeeding	Important for health/development, poor in Aboriginal children [33].		●	●	●					Parental questionnaire (CHS items 37,39) collated at 12 months [34].
		Body mass index	Childhood obesity is increasing. Home visiting interventions have had impact, although not sustained over time [35].						●		●	Child weight and height; BMI = kg/m2.
		Child development	Studies reporting significantly lower levels of performance for Indigenous children compared to their non-Indigenous counterparts on cognitive and language tasks at school entry [36, 37].					●			●	Griffiths child developmental assessment [14].
		Child vocabulary development									●	Peabody Picture Vocabulary Test – 4th Edition [38].
Secondary Outcomes	Child	Child birthweight	Low birth weight more prevalent in Aboriginal children [33].	●								Birth weight recorded in perinatal statistics.
		Child health	Child health is associated with health in later life [39].		●	●	●	●	●	●	●	General health (CHS item 97) [34]; Paediatric assessment (18 months).
		Child dental health	Dental disease is an important cause of potentially preventable hospitalisations particularly for young Aboriginal children age 0–4 [40].								●	Dental assessment (dmft: decayed, missing and filled primary teeth); Significant Caries Index (SiC).
		Illnesses and injury	Aboriginal children have increased hospital admissions for respiratory illness, ear disease, gastroenteritis [41, 42].		●	●	●	●	●	●	●	Parental questionnaire; Paediatric assessment (18 months); Hospital data collections. Collated at 24 and 48 months.
		Age at first solids	Disadvantaged mothers more likely to introduce solids too early [43].			●	●					Parental questionnaire (CHS items 48–49), collated at 12 months) [34].
		Age appropriate immunisation	Contact with health professionals is influential in immunisation [44].			●	●	●			●	Parental questionnaire validated by Child Personal Health Record (Blue Book) [45].
	Mother	Maternal enablement	Enabling mother is a key principle of SNHV [10, 46, 47].		●							Modified Patient Enablement Instrument (Groups A and B only) [48].
		Parental child developmental enablement	Parental enablement of their child’s development is a key purpose of the intervention.			●	●	●	●			Modified Patient Enablement Instrument (Groups A and B only) [48].
		Knowledge of SIDS risk factors	Disadvantaged families less likely to act to reduce risk of SIDS [49].	●	●							Parental questionnaire [50].
		Maternal health	Associated with child health.	●	●							12-Item Short Form Health Survey (SF-12) [51].
		Maternal smoking	Risk factor for adverse perinatal outcomes [33].	●	●	●	●	●	●	●	●	Fagerstrom test for nicotine dependence [52].
	Family	Maternal social support	Positively influences families, parents, children [53].	●	●	●	●	●	●	●	●	Parental questionnaire (CHS items 191–196) [34].
		Family functioning	Affects health and wellbeing of children [53].	●	●	●	●	●	●	●	●	McMaster Family Assessment Device [54].
	Home	Home environment	Stimulating environment associated with infant development [55].				●		●	●		HOME Inventory [56].
		Household smoking	Increases risk of child respiratory problems [57].	●	●	●	●	●	●	●	●	Parental questionnaire (CHS items 291–294) [34].
	Service use	Use of and satisfaction with services	Parenting programs are effective in improving child behaviour [58].	●	●	●	●	●	●	●	●	Parental questionnaire (CHS items 15, 18, 178–189, 285, 290) [34].
		Mother satisfaction with home visiting service	Mothers are more likely to make use of services that are accessible and acceptable [59].		●	●	●	●	●			Parental questionnaire (Modified PSQ-18 Groups A and B only) [50]; Program retention.

### Statistical methods

Microsoft Access is used to manage the data collection. The data will be extracted and analyses conducted using the latest version of SPSS. Descriptive analyses will be used to profile the characteristics of each Group. Although stratification will be applied during the recruitment processes for Group B, it is recognised that the three groups may differ on the distribution of demographic and risk factors impacting on treatment effects. To account for group differences in baseline demographic and risk profiles, propensity score analysis will be used to balance the differences in groups before treatment [28, 29].

Families recruited into Groups A and B are retained in the study regardless of whether they continued to receive the intervention. All analyses will be intention to treat. Comparison of the adjusted treatment effects for primary outcomes (duration [number of weeks] of breastfeeding, child development and educational development) will be made using t-tests, comparing outcomes of (1) Aboriginal children who did (Group A) and did not receive SNHV (Group C), and (2) Aboriginal and non-Aboriginal children receiving SNHV (Group A compared with Group B). Patterns of difference in secondary outcomes between the three groups will be analysed using Chi-Square test for proportions (categorical data) and ANOVA (continuous data), together with 95% confidence intervals. Multilevel analyses will be used for analyses of secondary outcomes collected at multiple time points (e.g., maternal health), with time at level 1 and the repeated outcome at level 2. Multiple regression analyses will be used to identify the impact of secondary measures as potential mediators or moderators of effects on the primary outcomes, for example, the impact of social support on the duration of breastfeeding, and the impact of the quality of the home environment and maternal health on child development.

## Discussion

This study is the first Australian trial of the effectiveness of sustained nurse home visiting for families of Aboriginal infants, and one of few conducted worldwide with Indigenous communities. Comparison with an historical non-intervention cohort of Aboriginal infants will allow assessment of the effectiveness of the intervention compared with usual care. In addition, enrolment of a contemporary study cohort of families of non-Aboriginal infants who also received sustained nurse home visiting provided by the same local health service provider in the same community will allow assessment of whether this intervention can ‘close the gap’ that exists in the health and development of Australian Indigenous and non-Indigenous children.

The intervention was based in the local public community health service and utilised the mainstream child and family health nurses and social workers, with the addition of Aboriginal Health Workers for the Aboriginal families receiving the intervention. By trialing the effectiveness of mainstream service provision for this population, the program is more likely to be replicable at the whole of population level than programs relying on provision by only Indigenous staff, who are considerably underrepresented in Australian child and family health professions.

The research is being conducted in partnership with the local service providers and Aboriginal community, who will support the communication of the study results through professional and community events, policy briefings and joint publications.

A significant limitation of the study was the number of families recruited to participate. Participation was limited by the maximum caseload that could be assigned to the number of trained program nurses. This limitation on recruitment numbers was further exacerbated by poorer than predicted retention rates in the intervention cohorts, and the Aboriginal intervention cohort in particular. Unfortunately, intervention participation could only be maintained for those who continually resided in the local area for the duration of the study, unlike the historical non-intervention group whose participation was not associated with an intervention. As has been the case with other home visiting research [30], attrition mainly occurred early in the research, predominantly in the antenatal period and the first month post-birth. This loss of participants was particularly high for the Aboriginal intervention group (Group A), with an overall loss of 30% of participants at those times. There is evidence [31] that in urban locations the Indigenous population is more mobile than the non-Indigenous population, and that Indigenous women are most mobile in young adulthood up to 30 years of age; the main age group in this study. The loss of participants in the perinatal period may reflect such mobility. The much higher retention rate in the historic cohort (80% at child-age 2 years) may also reflect postnatal study recruitment, rather than antenatal recruitment as in the case of the intervention groups, and therefore may not be fully indicative of the number of Aboriginal women presenting for antenatal care in the recruiting hospital. The low rates of participation at each data point may reduce the power of the study to detect small and moderate effects of the intervention. This may be moderated by the collection of the same data items at multiple data points, which should facilitate imputation of values for data points missed by individual participants and the use of multilevel analyses, however, this will remain a limitation for cross-sectional analyses.

Inferences about outcome differences between the Aboriginal and non-Aboriginal intervention groups may also be affected by the differences in the demographic and risk profile of the participating families. It was possible to match Groups A and B on maternal age, but not on suburb of residence, with Group A (like historic Group C) more likely to live in low socio-economic areas. There were also considerable issues of inconsistency in the routine Safe Start [20] assessment conducted by antenatal clinic midwives in either the asking, or recording of risk factors and vulnerabilities between the groups. Group B (non-Aboriginal intervention group) were much less likely to have an answer recorded regarding whether they had been abused as a child, or about mental health issues, whilst Group A (Aboriginal intervention group) were less likely to have data recorded about substance misuse. There also seems to have been a temporal change in recording of family violence with both current groups (A and B) being less likely to have these data recorded than the historic group (C Aboriginal non-intervention group). Propensity scoring will be used to correct recorded differences. For the purposes of identifying families to be offered the intervention, however, the assumption was made that non-recording of the vulnerability factor was an indicator of the absence of the risk. It is not possible to know whether the mother was not asked or whether she was asked and the factor was absent and hence not recorded.

The ‘Bulundidi Gudaga’ trial has been designed to provide Australian evidence of whether a sustained nurse home visiting intervention, adapted to meet the needs and preferences of an urban Aboriginal population and delivered by local public community health services, can improve outcomes for Aboriginal children and their families, and close the gap between them and their non-Aboriginal contemporaries. Although limited by the sample size and study retention and participation rates, it is envisaged that the comprehensive, culturally adapted intervention will support significant child health and development improvements that are sustained beyond the 2 year intervention to child age 4 years.

## Abbreviations

BMI: Body Mass Index
CHS: Child Health Survey
GQ: Griffiths Quotient
HIA: Health Impact Assessment
HOME: Home Observation for Measurement of the Environment
MECSH: Maternal Early Childhood Sustained Home-visiting
NSW: New South Wales, Australia
OECD: Organisation for Economic Co-operation and Development (OECD)
PSQ: Patient Satisfaction Questionnaire
SIDS: Sudden Infant Death Syndrome
SNHV: Sustained Nurse Home Visiting
